# Discrimination of Hexane Isomers by Temperature Swing Adsorption in a Rigid Aluminum Metal–Organic Framework

**DOI:** 10.1021/acsmaterialslett.6c00119

**Published:** 2026-03-11

**Authors:** Feng Xie, Liang Yu, Trevor Jenkins, Fu-An Guo, Shenfang Li, Timo Thonhauser, Hao Wang, Jing Li

**Affiliations:** † Department of Chemistry and Chemical Biology, Rutgers University, 123 Bevier Road, Piscataway, New Jersey 08854, United States; ‡ Hoffmann Institute of Advanced Materials, 47891 Shenzhen Polytechnic University, 7098 Liuxian Boulevard, Shenzhen, Guangdong 518055, P.R. China; § Department of Physics and Center for Functional Materials, 8676 Wake Forest University, 1834 Wake Forest Road, Winston-Salem, North Carolina 27109, United States; ∥ Department of Information Display, Kyung Hee University, 26 Kyungheedae-ro, Dongdaemun-gu, Seoul 02447, Republic of Korea

## Abstract

The efficient separation of alkane isomers with similar physicochemical properties remains a persistent challenge for the petrochemical industry. Adsorptive separation using metal–organic frameworks (MOFs) offers an energy-efficient alternative to conventional distillation. Herein, we report temperature swing discrimination of hexane isomers with different degrees of branching using MIL-120, a rigid aluminum pyromellitate-based MOF. MIL-120 features uniform one-dimensional channels with an aperture of ∼5.5 Å. At 30 °C, it selectively adsorbs linear and monobranched hexanes while excluding the dibranched isomer. Upon heating to 120 °C, both mono- and dibranched isomers are completely excluded, whereas linear hexane remains strongly adsorbed. Breakthrough experiments validate the temperature swing separation performance. Adsorption heat analysis combined with ab initio calculations provides a quantitative measure of distinct differences in adsorption enthalpies, binding energies, and diffusion barriers responsible for the observed separation efficiency, highlighting the potential of this MOF for efficient separation of alkane isomers via temperature swing adsorption.

## Graphical abstract

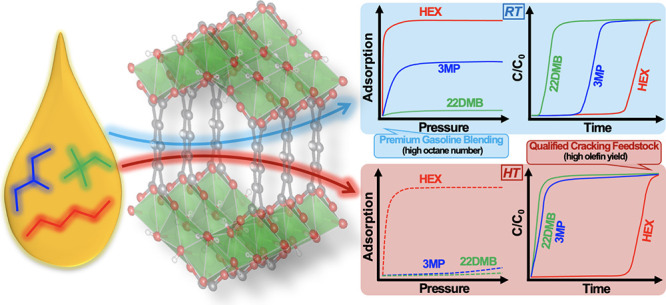

The separation of alkane isomers is of central importance to the petrochemical industry, as it yields qualified cracking feedstocks for olefin manufacture as well as premium gasoline with high octane numbers.^1,2^ Linear and monobranched alkanes are preferred feedstocks for steam cracking to produce ethylene, whereas more branched isomers possess higher octane numbers and are therefore desirable components in gasoline blends.^3,4^ Achieving efficient and complete separation of linear, monobranched, and dibranched isomers for these distinct end uses remains a technically challenging industrial process. An improved hexane separation strategy that selectively isolates branched isomers while recycling linear isomers back to the cracking reactor would substantially enhance process efficiency. Moreover, performing such separations at elevated temperatures compatible with cracking conditions would significantly reduce the energy consumption associated with cooling and reheating steps.^5,6^

At present, heat-driven distillation dominates industrial alkane isomer separation; however, its inherently high energy demand and capital cost motivate the development of alternative or complementary energy-efficient technologies.^7,8^ Selective adsorption using porous materials provides a promising route to reduce energy consumption and operating costs.^9,10^ Because hexane isomers are chemically inert and exhibit nearly identical physicochemical properties, differences in molecular size and shape constitute the primary basis for separation via adsorption. Consequently, effective discrimination among these isomers imposes stringent requirements on the pore aperture and geometry of the adsorbent. Zeolite 5A, with a pore aperture of approximately 5 Å, serves as a benchmark adsorbent material and is capable of fully separating linear hexane from branched isomers.^11^ However, its relatively low adsorption capacity limits separation efficiency, and it lacks the ability to distinguish between mono- and dibranched isomers. In contrast, ZSM-5, featuring channels of approximately 5.5 Å, can discriminate monobranched from dibranched isomers, albeit with limited uptake.^12^ These limitations underscore the need for advanced adsorbents that combine high capacity with precise molecular discrimination.

Metal–organic frameworks (MOFs) are particularly attractive candidates for addressing these challenges due to their high porosity and tunable pore structures.^13–15^ Their structural versatility has enabled successful separations of molecules with minimal differences in size and other properties. Advances in reticular chemistry allow precise control over pore size and shape, facilitating the rational design of MOFs with targeted selectivity toward alkane isomers with varying degrees of branching.^16–20^ Over the past decades, numerous MOFs have been investigated for alkane isomer separation.^1,2,21–23^ For example, Fe_2_(BDP)_3_ accommodates linear, mono-, and dibranched hexanes through a thermodynamically driven process, exhibiting high adsorption capacities but relatively low separation selectivity.^24^ On the other hand, rigid MOFs capable of fully separating hexane isomers via molecular sieving remain rare,^20,23,25–28^ particularly under industrially relevant conditions.

Herein, we report MIL-120 as a cost-effective MOF capable of highly efficient and complete discrimination of hexane isomers over a wide temperature range. MIL-120 features rigid one-dimensional (1D) channels with a hexagonal cross section. At room temperature, it adsorbs linear and monobranched alkanes while nearly completely excluding dibranched isomers. At elevated temperature, it excludes both mono- and dibranched isomers while selectively adsorbing linear hexane. The highest nHEX/3MP and nHEX/22DMB uptake ratios are achieved for MIL-120 over all previously reported high-performing MOFs. Column breakthrough experiments confirm complete separation of hexane isomers at room temperature and selective exclusion of branched isomers at elevated temperature. Results from heat flow measurements and ab initio calculations provide mechanistic insight into the host–guest interactions and elucidate the temperature swing effect of the observed separation efficiency.

MIL-120 was synthesized hydrothermally using aluminum nitrate and pyromellitic acid, following a previously reported procedure.^29^ Its crystal structure is built from edge-sharing aluminum-oxide octahedra that assemble into infinite Al–OH-Al zigzag chains by alternating between *cis* and *trans* conformations (Figure 1a). These inorganic chains are bridged by pyromellitate linkers to generate a three-dimensional (3D) framework containing 1D channels parallel to the crystallographic *c*-axis. The resulting pores feature a hexagon-shaped cross section of 4.8 × 5.4 Å (Figure 1b &1 **c**). The phase purity and crystallinity of MIL-120 were confirmed by powder X-ray diffraction (PXRD) analysis, with the experimental patterns of both as-made and activated samples in good agreement with the simulated pattern (Figure S1). Thermogravimetric analysis (TGA) of the as-made compound, Al_4_(OH)_8_(C_10_O_8_H_2_)·5H_2_O, shows a gradual mass loss of approximately 15 wt % upon activation up to ∼ 100 °C, which is attributed to the removal of guest water molecules from the pore channels (Figure S2).

**Figure 1:**
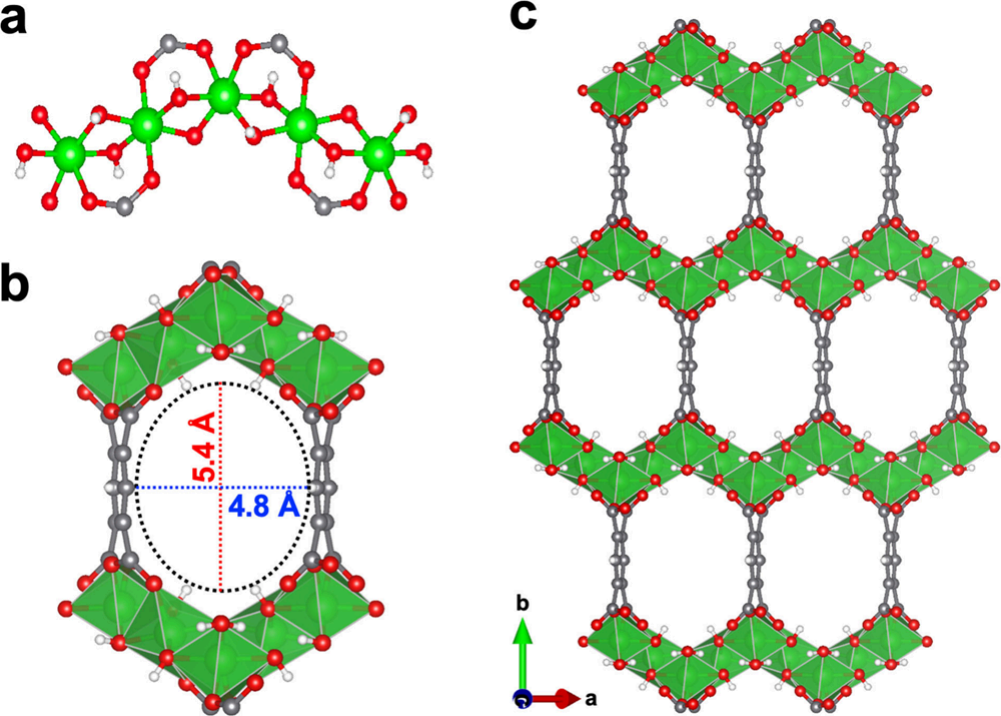
(**a**) Al–OH-Al chains constituting the inorganic backbone of MIL-120. (**b**) The cross section of the 1D pore channel viewed along the crystallographic *c* axis, highlighting its size of 5.4 × 4.8 Å. Green polyhedra represent aluminum coordination environments. (**c**) Crystal structure of the guest-free MIL-120 viewed along the *c* axis, showing 1D channels with a hexagonal cross section. Green, red, gray, and white spheres represent Al, O, C, and H atoms, respectively.

The porosity of activated MIL-120 was evaluated by carbon dioxide adsorption at 195 K, yielding a Brunauer–Emmett–Teller (BET) surface area of 383 m^2^ g^–1^ and a narrow pore size distribution centered at approximately 5.5 Å, as determined using the Horvath–Kawazoe (H–K) model (Figure 2a). The well-matched pore aperture of MIL-120 motivated our investigation of its potential for the adsorptive separation of hexane isomers, with their kinetic diameters ∼ 4.5 Å for linear hexane, ∼ 5.0–5.5 Å for monobranched isomers, and ∼ 6.2 Å for dibranched isomers (Table S1). Single-component vapor adsorption experiments were therefore conducted using linear *n*-hexane (nHEX), monobranched 3-methylpentane (3MP), and dibranched 2,2-dimethylbutane (22DMB) at 30 and 120 °C. At 30 °C, MIL-120 exhibits high uptake of nHEX and moderate uptake of 3MP, with capacities of 135 and 87 mg g^–1^, respectively (Figure 2b), while the uptake of 22DMB is very low. Notably, the adsorption capacity of MIL-120 for nHEX is competitive with those of previously reported high-performing MOF adsorbents capable of discriminating linear and branched hexanes, including Fe_2_(BDP)_3_, Ni­(4-PyC)_2_,^25^ CAU-21-ODB,^30^ CAU-10-H,^31^ Ca-tcpb,^22^ HIAM-203,^32^ HIAM-601,^20^ and Zr-abtc^23^ under comparable conditions (Table S3).

**Figure 2:**
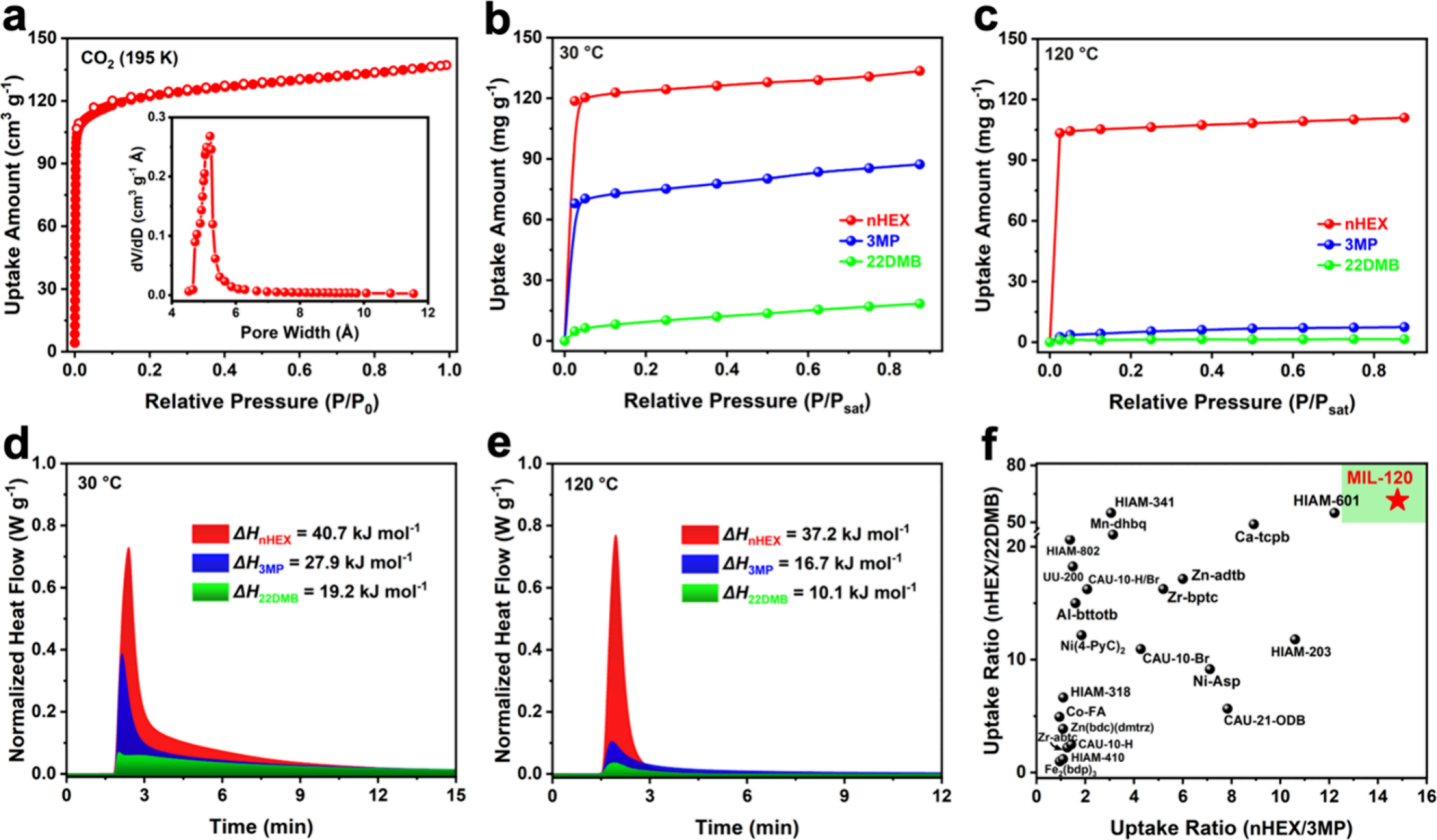
(**a**) CO_2_ sorption isotherms of MIL-120 at 195 K and corresponding pore size distribution derived from the H–K model. (**b**) Single-component vapor adsorption isotherms of nHEX, 3MP, and 22DMB on MIL-120 at 30 °C and (**c**) 120 °C. (**d**) Heat flow traces and corresponding adsorption enthalpies (*ΔH* < 0) for hexane isomer adsorption on MIL-120 obtained from thermogravimetric DSC measurements at 30 °C and (**e**) 120 °C. (**f**) Comparison of nHEX/3MP and nHEX/22DMB uptake ratios for representative high-performing MOF adsorbents reported to date. Uptake ratios were collected at different temperatures: 160 °C (Fe_2_(BDP)_3_); 150 °C (Ni-Asp, Zr-bptc, Zr-abtc, HIAM-203); 120 °C (MIL-120, Ca-tcpb, Mn-DHBQ); 100 °C (HIAM-341); 50 °C (PTA); and 30 °C (all other MOF adsorbents). Experimental temperatures and other details are provided in Table S3.

Upon increasing the temperature to 120 °C, MIL-120 retains a high uptake of nHEX (111 mg g^–1^), only slightly lower than that observed at 30 °C (Figure 2c). It shows fast adsorption kinetics for nHEX at both temperatures, as evidenced by steep dynamic uptake profiles and fast saturation behavior (Figures S3 & S4), indicative of strong host–guest interactions and low diffusion barriers. In contrast, adsorption of both 3MP and 22DMB becomes negligible at 120 °C, demonstrating effective exclusion of branched isomers under elevated temperatures (Figure 2c). These results highlight the ability of MIL-120 to achieve efficient temperature swing discrimination of hexane isomers as a function of molecular branching.

The temperature-dependent adsorption behavior observed in MOFs often arise from their framework flexibility or structural transformation upon guest loading.^22,27,32–37^ However, hydrocarbon-loaded PXRD patterns collected for activated MIL-120 after exposure to three hexane isomers and at different temperatures show no detectable structural changes (Figures S5 & S6), confirming the rigidity of the framework. Therefore, unlike previously reported flexible MOFs exhibiting temperature-dependent molecular-sieving based separation mechanism driven by structural transformations,^38–40^ the temperature swing adsorption observed here for MIL-120 does not exhibit apparent changes in its framework. Specifically, the decrease in adsorption uptake with increasing temperature reflects the shift of energy balance, whereby increased kinetic energy at elevated temperatures overcomes host–guest interactions.

To quantitatively evaluate the energetic landscape of hexane isomer adsorption in MIL-120, differential scanning calorimetry (DSC) measurements were conducted on activated MIL-120 samples during exposure to the three isomers at 30 and 120 °C. The adsorption enthalpies (*ΔH*) at 30 °C were determined to be 40.7 kJ mol^–1^ for nHEX, 27.9 kJ mol^–1^ for 3MP, and 19.2 kJ mol^–1^ for 22DMB (Figure 2d). At 120 °C, the corresponding adsorption enthalpies decrease to 37.2 kJ mol^–1^ for nHEX, 16.7 kJ mol^–1^ for 3MP, and 10.1 kJ mol^–1^ for 22DMB (Figure 2e). The relatively high adsorption enthalpy of nHEX at elevated temperature indicates persistent strong host–guest interactions, whereas the pronounced reduction in adsorption enthalpies for 3MP at higher temperature reflects significantly weakened interactions, consistent with its negligible uptake. For 22DMB, the low adsorption enthalpies at both 30 and 120 °C are consistent with its low uptake at these temperatures. These calorimetric results are in full agreement with the observed temperature swing adsorption behavior. Taken together with the high nHEX adsorption amount and highest nHEX/3MP and nHEX/22DMB uptake ratios, MIL-120 compares favorably with previously reported high-performing MOFs for the complete discrimination of hexane isomers (Figure 2f).

The results from single-component adsorption measurements suggest that MIL-120 is likely to be capable of achieving separation of all three hexane isomers at room temperature with moderate performance, while producing an high-purity optimum cracking feedstock at elevated temperature (120 °C) by selectively excluding both branched isomers from linear nHEX via molecular sieving. To experimentally validate this potential and quantify the separation performance, dynamic breakthrough experiments were conducted at different temperatures using a fixed-bed column packed with MIL-120 and fed with an equimolar ternary mixture of hexane isomers. The breakthrough profiles closely follow the adsorption hierarchy established from the single component vapor adsorption isotherms. At 30 °C, the dibranched 22DMB eluted first (at ∼ 5 min), followed by the monobranched 3MP (at ∼ 20 min), while linear nHEX is retained in the column for ∼ 43 min, resulting in complete separation of all three isomers (Figure 3a). On the other hand, at 120 °C, both branched isomers (3MP and 22DMB) eluted immediately at the onset of the experiment, indicating minimal retention and confirming their effective exclusion by MIL-120 (Figure 3b). Under this condition, the linear nHEX is initially retained within the column and begins to elute only after ∼ 70 min, nearly twice as long as that at 30 °C.

**Figure 3:**
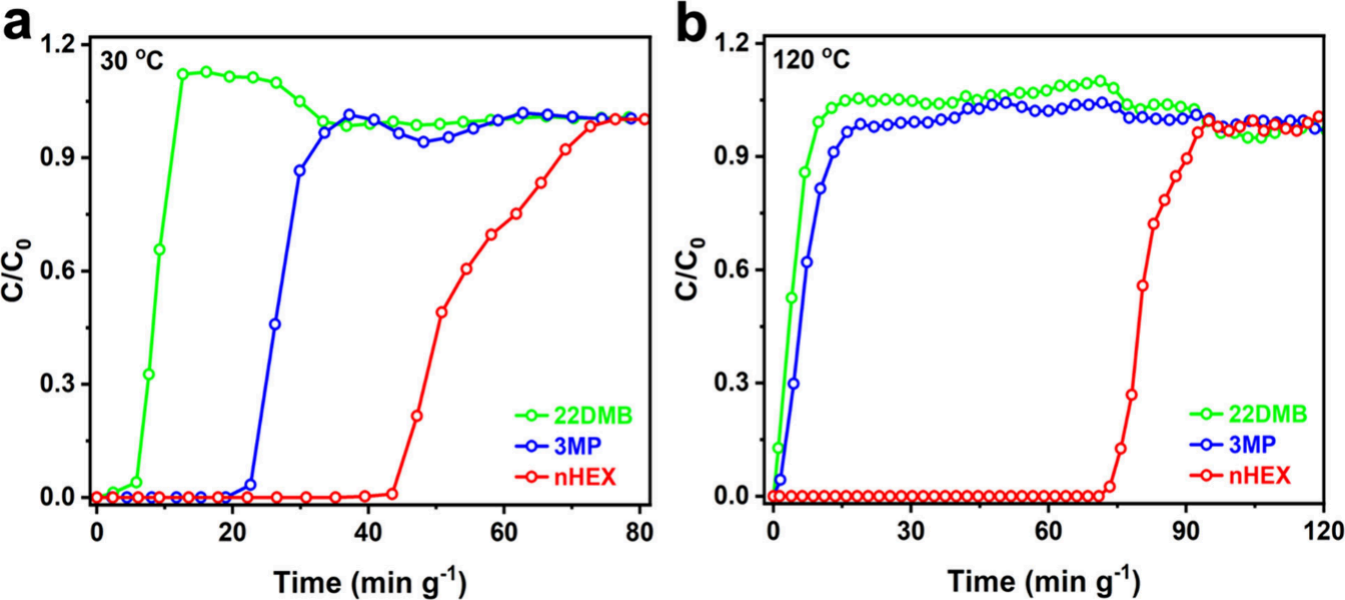
(**a**) Column breakthrough curves for an equimolar nHEX/3MP/22DMB ternary mixture on MIL-120 at 30 °C and (**b**) 120 °C.

To gain molecular-level insight into the host–guest interactions governing hexane adsorption in MIL-120, ab initio calculations were performed on the system. The study began with full structural optimization of the MIL-120 unit cell, followed by the introduction of individual hexane isomers to identify the most energetically favorable adsorption configurations. For all three guests, the lowest-energy structures place the molecules near the center of the pore, without covalent binding to the framework. Charge density difference analyses reveal that adsorption is dominated by van der Waals interactions and weak hydrogen bonding between the hexane molecules and hydroxyl groups associated with the aluminum nodes (Figures 4a-4c & S7). The calculated binding energies corroborate the experimentally observed preferential adsorption trends of nHEX > 3MP > 22DMB. Linear nHEX, monobranched 3MP, and dibranched 22DMB exhibit binding energies of 1.43, 1.36, and 0.97 eV, respectively (Figure 4d). These binding energies correspond to electronic energies at absolute zero (0 K) and are therefore not directly comparable to adsorption enthalpies obtained from DSC experiments at given temperatures. Using the phonon data reported in a recent study on the same molecules,^41^ we estimated the corresponding binding free energies at 30 °C to be 0.67, 0.54, and 0.17 eV for nHEX, 3MP, and 22DMB, respectively, in good agreement with the trend of experimental thermodynamic data.

**Figure 4:**
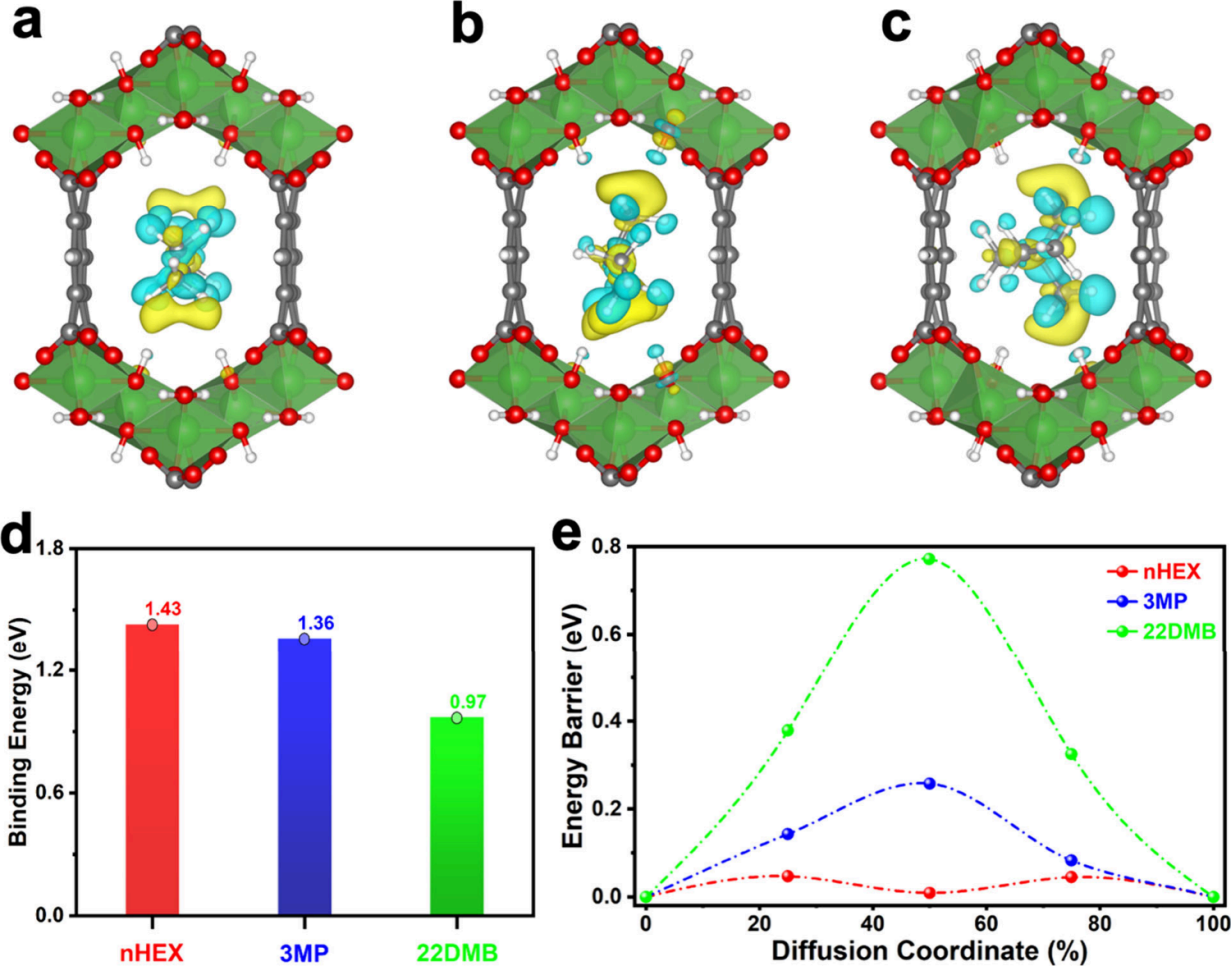
(**a**) Induced charge density for interactions of nHEX, (**b**) 3MP, and (**c**) 22DMB at their optimized binding sites in MIL-120, illustrating induced charge rearrangement upon binding. Yellow and blue isosurfaces represent charge accumulation and depletion, respectively, with an isosurface value of 0.001 e/Å^3^. (**d**) Calculated binding energies and (**e**) diffusion barriers of the three hexane isomers within MIL-120. Diffusion coordinates from 0% to 100% correspond to fully optimized guest configurations in neighboring unit cells.

To further elucidate kinetic contributions to adsorption, diffusion barriers for each guest were calculated using the climbing nudged elastic band (cNEB) method. The resulting barrier heights are 0.05 eV for nHEX, 0.26 eV for 3MP, and 0.77 eV for 22DMB (Figure 4e). These results indicate facile diffusion of nHEX through the MIL-120 channels, whereas diffusion of branched isomers—particularly 22DMB—is significantly hindered. According to the Arrhenius equation, diffusion of 3MP and 22DMB is suppressed by factors of approximately 3 × 10^3^ and 1 × 10^12^, respectively, relative to nHEX at 30 °C. Thus, transport of the branched isomers within the MIL-120 channels is strongly impeded, and 22DMB is effectively excluded compared to nHEX, in agreement with the experiment observations. The combination of weaker binding interactions and a substantially higher diffusion barrier for 22DMB provides a mechanistic explanation for its negligible uptake. Overall, the computational results demonstrate that selective adsorption in MIL-120 is governed by a synergy of thermodynamic and kinetic factors, with stronger binding and lower diffusion barriers favoring linear nHEX and steric constraints effectively suppressing adsorption of branched isomers, in excellent agreement with experimental observations.

The recyclability and chemical robustness of adsorbents are critical considerations for practical applications. To evaluate the recyclability of MIL-120, ten consecutive cycles of dynamic sorption experiments were performed. As shown in Figure S8, the nearly overlapping sorption profiles across all cycles demonstrate excellent regeneration capability and cycling stability. In addition, PXRD patterns collected after repeated sorption cycles and after exposure to various chemical environments remain unchanged, confirming the high structural stability and chemical durability of MIL-120 (Figure S9). Collectively, these results underscore the promise of MIL-120 as a low-cost, robust, and efficient adsorbent for practical separation processes.

In summary, the selective separation of alkanes with different degrees of branching remains a critical challenge in the petrochemical industry for the production of high-octane gasoline blends and high-quality cracking feedstocks. This study demonstrates that MIL-120, a cost-effective and rigid aluminum pyromellitate-based microporous MOF with high framework stability, offers substantial potential to address this challenge. Owing to its well-matched pore aperture, MIL-120 preferentially adsorbs linear and monobranched alkanes while nearly excluding dibranched isomers at room temperature, and achieves complete exclusion of branched isomers while selectively retaining linear hexane at elevated temperature. Column breakthrough experiments confirm complete separation of hexane isomers at room temperature with moderate performance and selective retention of linear hexane while excluding all branched isomers under elevated temperature. Heat flow analysis combined with ab initio calculations provides mechanistic insight into host–guest interactions and elucidates the temperature swing effect governing selective adsorption. Collectively, these findings provide valuable insight for the rational design of advanced adsorbents for practical and energy-efficient alkane separations in the petrochemical industry.

## Supplementary Material



## Notes

Experimental and computational details, PXRD patterns, TGA curves, adsorption kinetics, hydrocarbon-loaded PXRD patterns, simulated calculations, recyclability and stability tests, supporting tables, and supporting references (PDF)
