# Supplementary material for: Discrimination of Hexane Isomers by Temperature Swing Adsorption in a Rigid Aluminum Metal–Organic Framework
Source: ACS Mater Lett. 2026 Mar 11;8(4):1252–8. doi: 10.1021/acsmaterialslett.6c00119 (PMC13058947; doi:10.1021/acsmaterialslett.6c00119)
Supplement: Supplementary file 1 [file tz6c00119_si_001.pdf]

Supporting Information

Discrimination of Hexane Isomers by Temperature Swing Adsorption in A Rigid Aluminum Metal-Organic Framework

Feng Xie,[a] Liang Yu,[b] Trevor Jenkins,[c] Fu-An Guo,[b] Shenfang Li,[b] Timo Thonhauser,[c] Hao Wang,[b,*] and Jing Li[a,d,*]

[a] Department of Chemistry and Chemical Biology, Rutgers University, 123 Bevier Road, Piscataway, New Jersey 08854, United States

[b] Hoffmann Institute of Advanced Materials, Shenzhen Polytechnic University, 7098 Liuxian Boulevard, Shenzhen, Guangdong 518055, P.R. China

[c] Department of Physics and Center for Functional Materials, Wake Forest University, 1834 Wake Forest Road, Winston-Salem, North Carolina 27109, United States

[d] Department of Information Display, Kyung Hee University, 26 Kyungheedae-ro, Dongdaemun-gu, Seoul 02447, Republic of Korea

* Correspondence to: wanghao@szpu.edu.cn (H.W.); jingli@rutgers.edu (J.L.)

This file includes:

Methods

Supporting Figures S1 to S9

Supporting Tables S1 to S3

References

Methods

Synthesis of MIL-120

All chemicals were used as received without further purification. MIL-120 was hydrothermally synthesized from a mixture of aluminum nitrate nonahydrate ($Al(NO_3)_3{\cdot}9H_2O$, 4.2 mmol) and 1,2,4,5-benzenetetracarboxylic acid or pyromellitic acid (H_4BTEC or PM, 1 mmol) in a aqueous solution (12 mL) with adding of sodium hydroxide (NaOH, 6.6 mmol). Then the resulting mixture was placed in a 23 mL Teflon steel autoclave and heated at 180 °C for 24 hrs. The resulted white powder product was centrifuged and dried in 60 °C oven.

Physical Characterizations

Powder X-ray diffraction (PXRD) measurements were performed on a Rigaku Ultima-IV automated diffraction system (Rigaku, Japan) using Cu-Kα radiation (λ = 1.5406 Å) in the 2 Theta range of 3-40° with a scan rate of 3° min^{-1}. The operating powder was 40 kV/44 mA. Thermogravimetric analysis of MIL-120 was performed on the TA Instrument Q5000 thermogravimetric analyzer. About 10 mg of the as-made sample was loaded onto a platinum pan and heated under nitrogen flux with a heating rate of 5 °C min^{-1} from room temperature to 700 °C. For the activated MIL-120, a fresh as-made sample was pre-activated at 150 °C in a vacuum oven for 3 hrs to remove guest water molecules, and then tranferred to the thermogravimetric analyzer for thermal stability testing under the same conditions as those used for the as-made sample. The BET surface area and pore size distribution determinations were measured by carbon dioxide sorption isotherm in a Micromeritics 3Flex adsorption analyzer (Micrometrics, USA) at 195 K. Prior to the gas sorption test, the fresh sample was activated at 150 °C for 6 hrs under dynamic vacuum condition before measurements.

Single-Component Vapor Adsorption Measurements

Single-component vapor adsorption isotherms measurements were performed on a homemade gravimetric adsorption unit modified from a TA Instrument Q50 thermogravimetric analyzer. Pure nitrogen was used as a carrier gas passing through a bubbler filled with pure liquid hexane isomer. The partial pressures of hexane isomer were

controlled by adjusting the blend ratio of pure nitrogen and mixed nitrogen with saturated isomer vapors. Adsorbed amount was monitored by the weight change of sample over the experiment period in the TA computer system. About 20 mg of MIL-120 sample was activated at 150 °C under nitrogen flow for 3 hrs to remove any residual guest molecules. The temperature was then cooled down to the designed temperature (30 and 120 °C), and another nitrogen flow passing through hexane isomer bubbler at room temperature was mixed with the pure nitrogen stream. The mixed gases stream was finally introduced to the adsorption chamber where the temperature can be controlled with the heating furnace and circulating water.

Recycling Sorption Test

The recyclability test of MIL-120 was performed on TA Instrument Q50 gravimetric adsorption analyzer for consecutive nHEX adsorption-desorption cycles. About 20 mg of MIL-120 sample was activated at 150 °C under nitrogen flow for 3 hrs before the experiments. After cooling down to 120 °C, another nitrogen flow passing through liquid nHEX bubbler was introduced to the adsorption chamber. After the adsorption reached equilibrium, the nHEX-adsorbed samples was desorbed at 150 °C for 1 hr to reactivate the MIL-120 sample for the following consecutive adsorption-desorption recycling test.

Heat Flow Measurements

The heat flows in the adsorption process were measured hexane isomers using a differential scanning calorimetry (DSC) system by TA instrument. Runs at the thermobalance were performed by feeding a flow stream of hexane isomer diluted with nitrogen onto about 10 mg of activated sample at the designated temperature (30 and 120 °C). Prior to measurements, the baseline was monitored under dry nitrogen flow at the same temperature, and then a nitrogen gas flow was introduced by bubbling the carrier gas in a saturator containing pure hexane isomer and the DSC signals were recorded. The scanning calorimetry curves were collected through real-time detection on the heat changes of the overall system inside the chamber during the adsorption process. The enthalpies (ΔH) were determined by the area (S) of integrating the heat change with respect to time according to the equation:

$$\Delta H = \frac{S \times m}{6 \times 10^{-2} \times n}$$

Here, ΔH is the enthalpies expressed in kJ mol^{-1}. S is the integrating area expressed in W·min g^{-1}, m is the amount of used sample expressed in g, and n is the amount of adsorbed hexane isomer expressed in mol.

Hydrocarbon-Loaded Powder X-ray Diffraction Analysis

PXRD coupled with vapor adsorption measurements were performed on a Rigaku Ultimate-IV diffraction system equipped with thermogravimetric analyzer for hydrocarbon adsorption. Typically, about 20 mg as-made MIL-120 was activated at 150 °C and cooled down to the designed temperature (30 and 120 °C). The flow of nitrogen passed through the hexane bubbler to introduce it into the adsorption chamber. After achieving the adsorption equilibrium, the real-time MIL-120 sample with vapor adsorption was quickly sent to PXRD system for the subsequent diffraction data collection.

Multicomponent Vapor Column Breakthrough Experiments

Breakthrough test was carried out in a lab-scale fix-bed reactor equipped with vapor generator. The mass of MIL-120 that was packed into the column (I.D. 4 mm x 300 mm) was nearly 0.4 g. The adsorbent was activated by heating to 150 °C for 3 hrs under helium purging (15 mL min^{-1}). The temperature was then cooled down to the designed temperature (30 or 120 °C), and another dry helium flow at a flow rate of 1 mL min^{-1} was bubbled through a mixture of hexane isomers (the vapor phase ratios were optimized to an equimolar mixture: 5.84 mL of nHEX, 4.12 mL of 3MP, and 2.57 mL of 22DMB for nHEX/3MP/22DMB ternary equimolar mixture) to pass through the adsorption bed. The outlet gas from the column was analyzed by using an online GC equipped with HP-PONA column and FID.

Computational Details

All the ab initio calculations were done using the VASP code.[1, 2] Van der Waals interactions were modeled using the non-local correlation of the vdW-DF functional.[3-6] For pseudopotentials, we used the standard projected augmented wave (PAW) potentials along with a kinetic-energy cutoff of 600 eV. All adsorption calculations used a 3 x 1 x 1 supercell of the MOF with a single guest molecule present. Relaxation calculations

optimized atomic positions and cell dimensions, stopping when the total forces on all atoms were less than 0.005 eV/Å. We calculated interaction energies by subtracting energies of the fully optimized empty MOF and the fully optimized guest molecule from the energy of the entire adsorption complex:

$$E_b = E_{ads} - E_{MOF} - E_{guest}$$

Calculations of the diffusion barrier were performed using the climbing nudged elastic band method (cNEB).[7] This involved structural optimizations of the adsorption system, placing the guest molecule at different coordinates along the pore axis, and comparing the energies of each configuration. All cutoffs and convergence criteria for these calculations were the same as in the previous optimizations. To visualize sorbate-sorbent interactions, we calculate the induced charge density, i.e., the charge rearrangement upon bond formation, as the difference of the density between total and individual fragments.

Supporting Figures

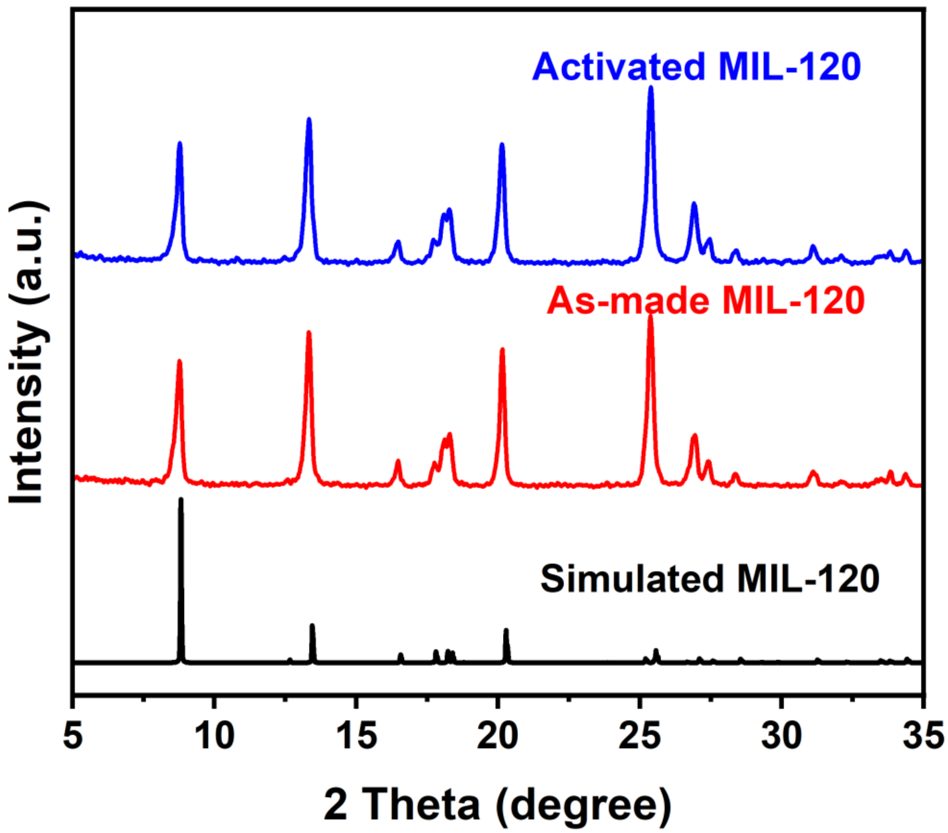

Figure S1. Powder X-ray diffraction (PXRD) patterns of the as-made and activated samples of MIL-120 compared with the simulated one.

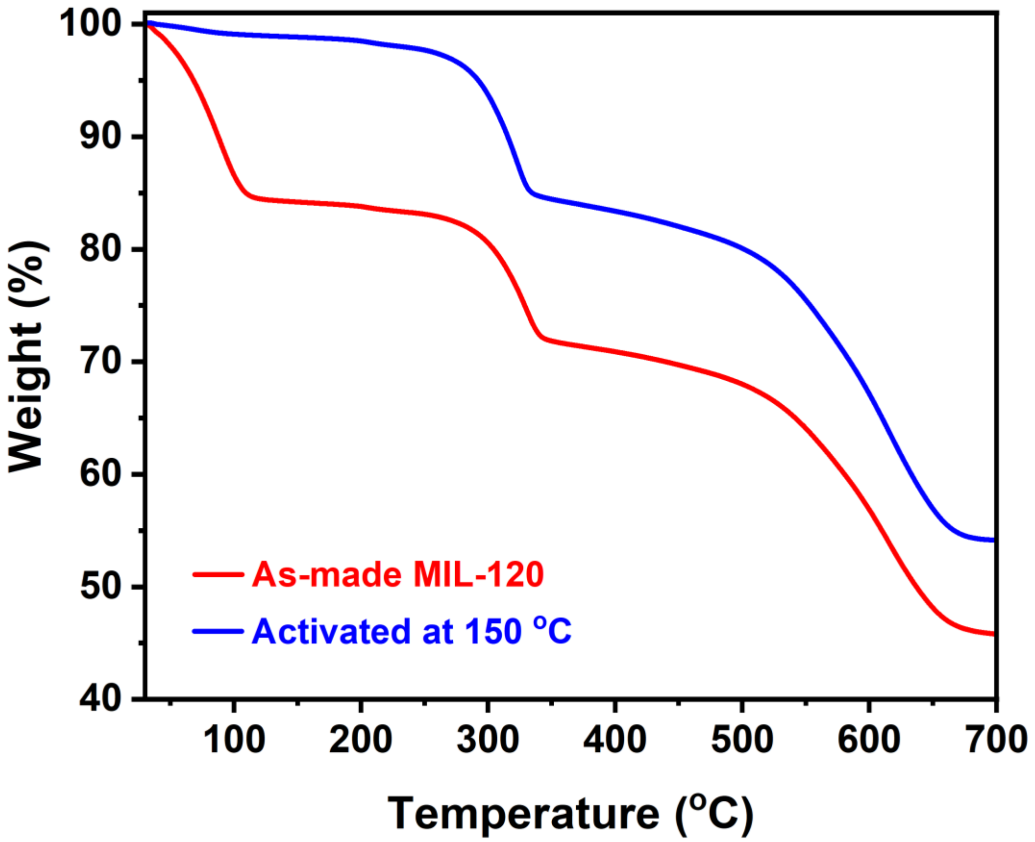

Figure S2. Thermogravimetric (TG) curves of the as-made and activated MIL-120 samples collected under nitrogen with a heat rate of 5 °C min^{-1}.

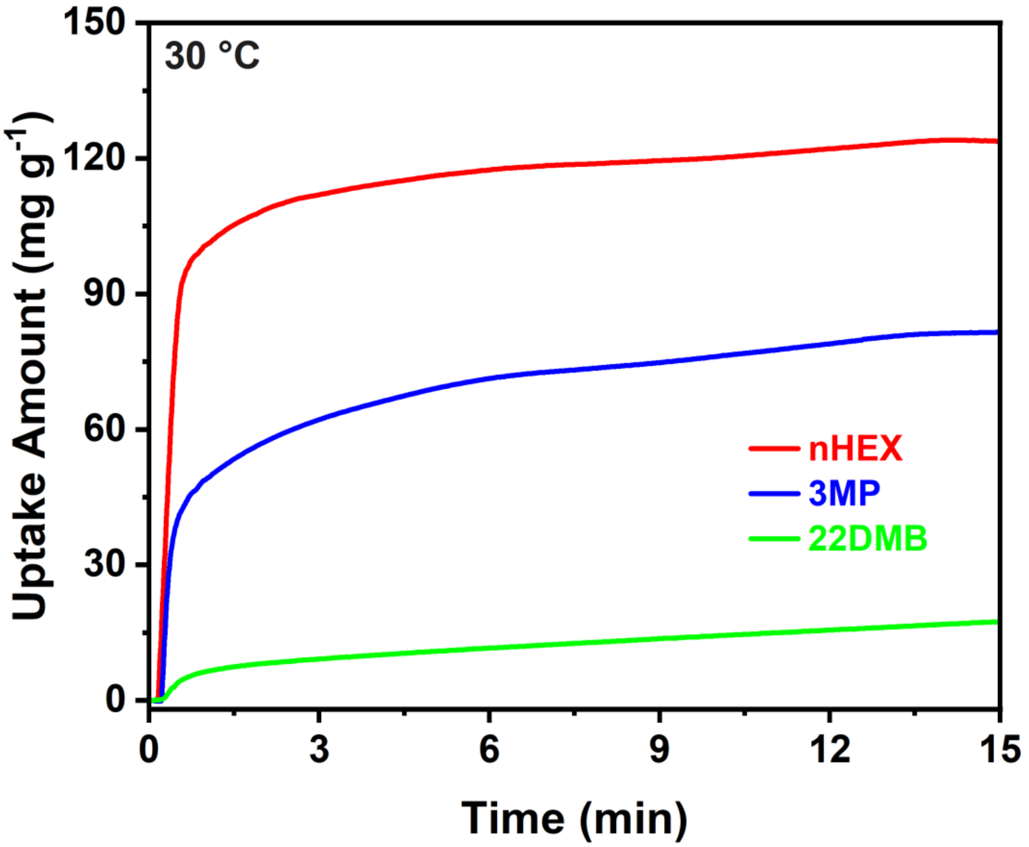

Figure S3. Adsorption kinetics of hexane isomer vapors on MIL-120 at 30 °C.

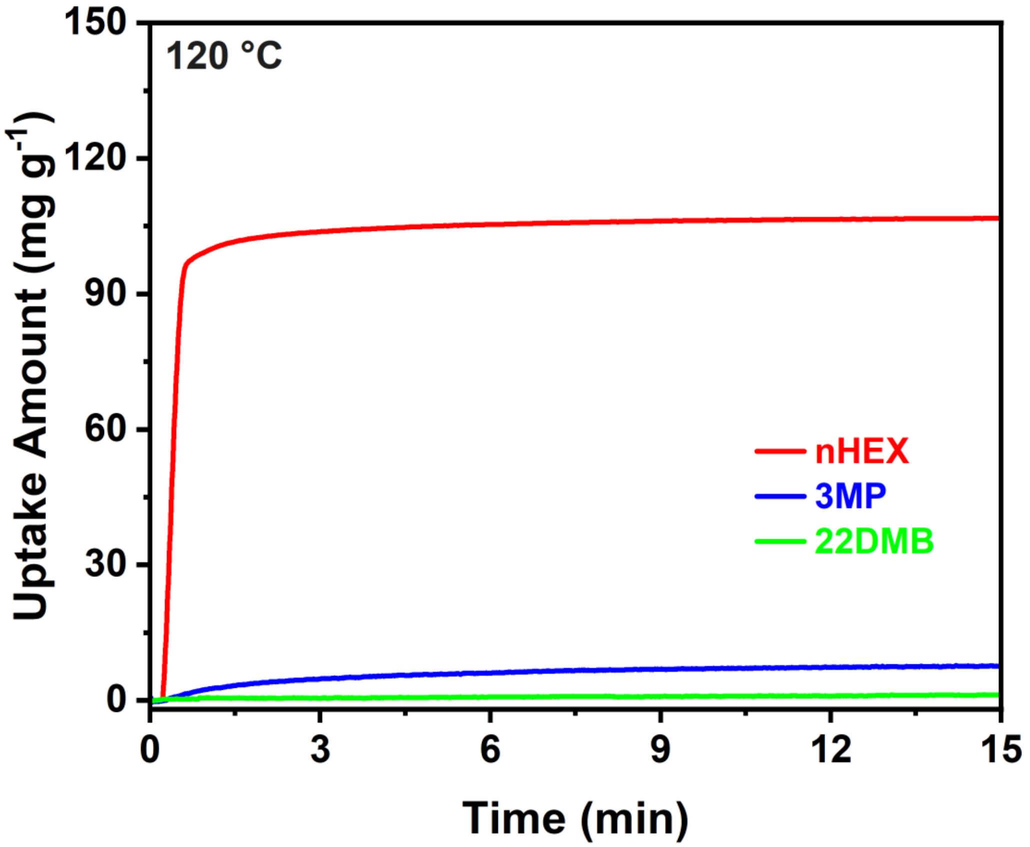

Figure S4. Adsorption kinetics of hexane isomer vapors on MIL-120 at 120 °C.

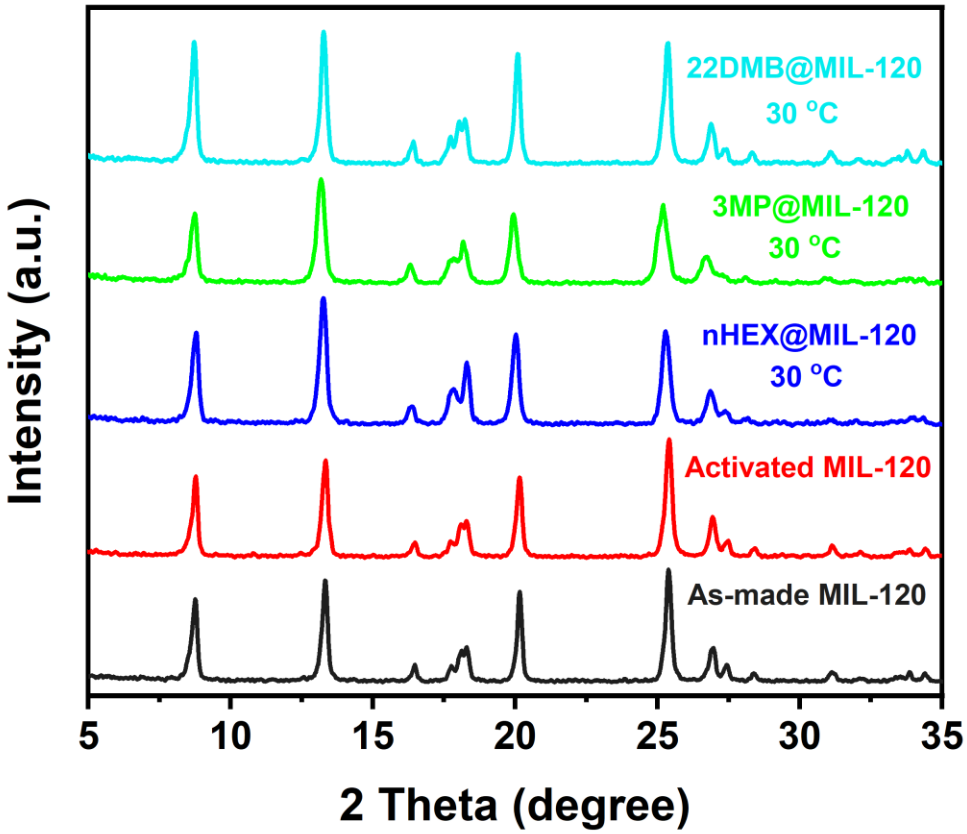

Figure S5. PXRD patterns of hydrocarbons-adsorbed MIL-120 at 30 °C compared with as-made and activated ones.

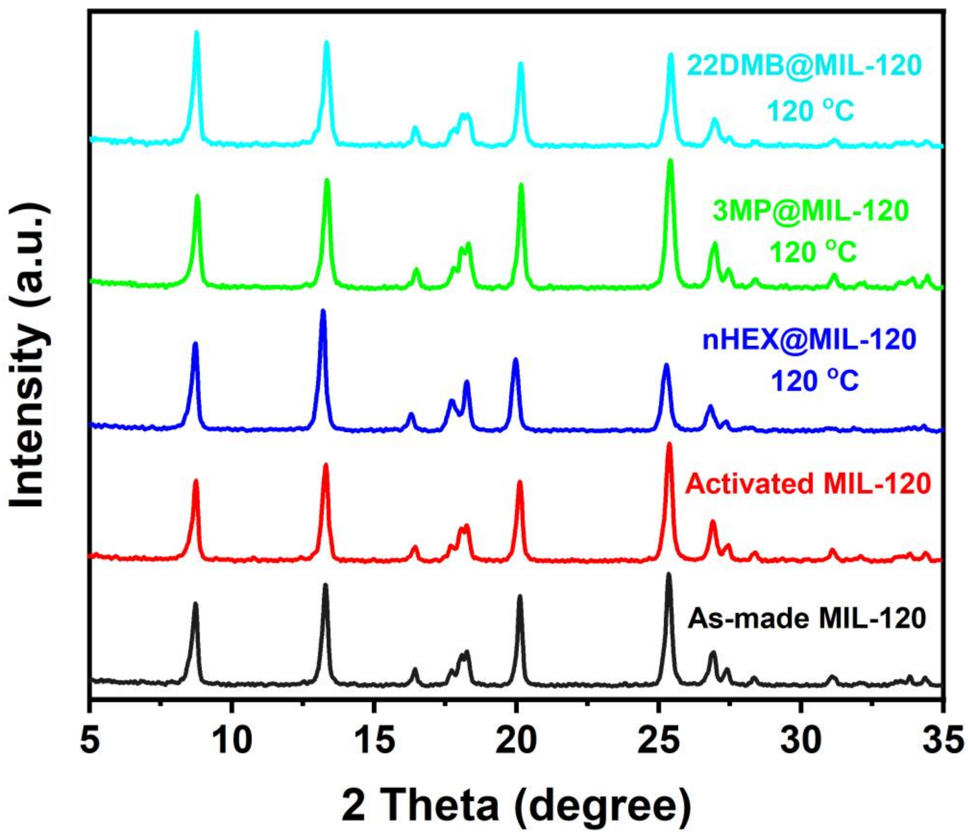

Figure S6. PXRD patterns of hydrocarbons-adsorbed MIL-120 at 120 °C compared with as-made and activated ones.

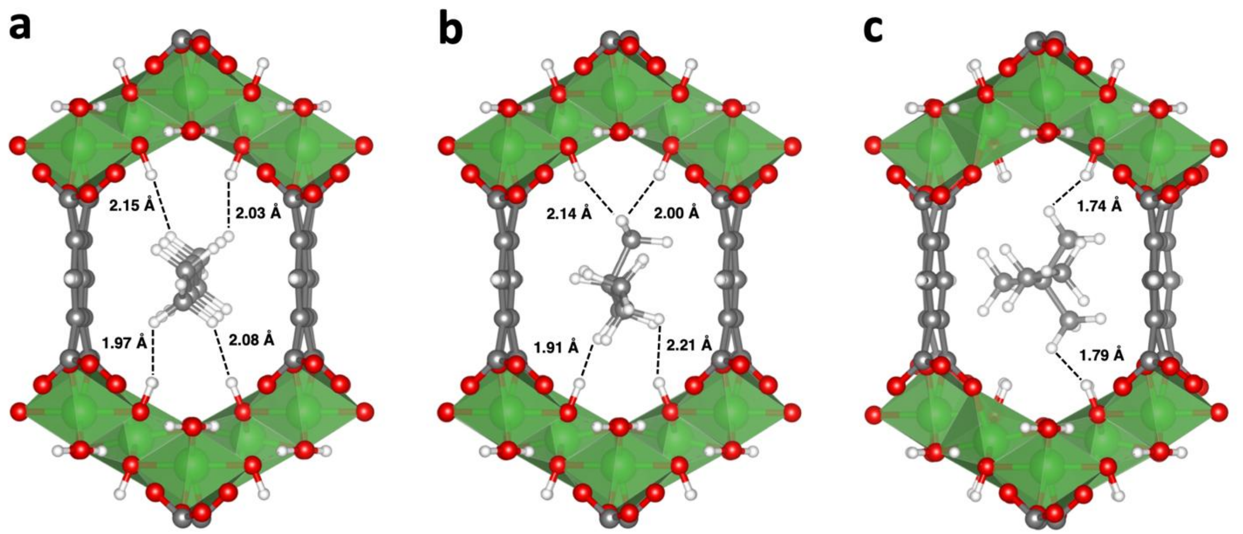

Figure S7. (**a**) Calculated binding configurations for nHEX, (**b**) 3MP, and (**c**) 22DMB with binding distances labeled within MIL-120. Connections between oxygen and hydrogen atoms are the result of hydrogen bonds, while carbon and hydrogen connections are due to van der Waals interactions.

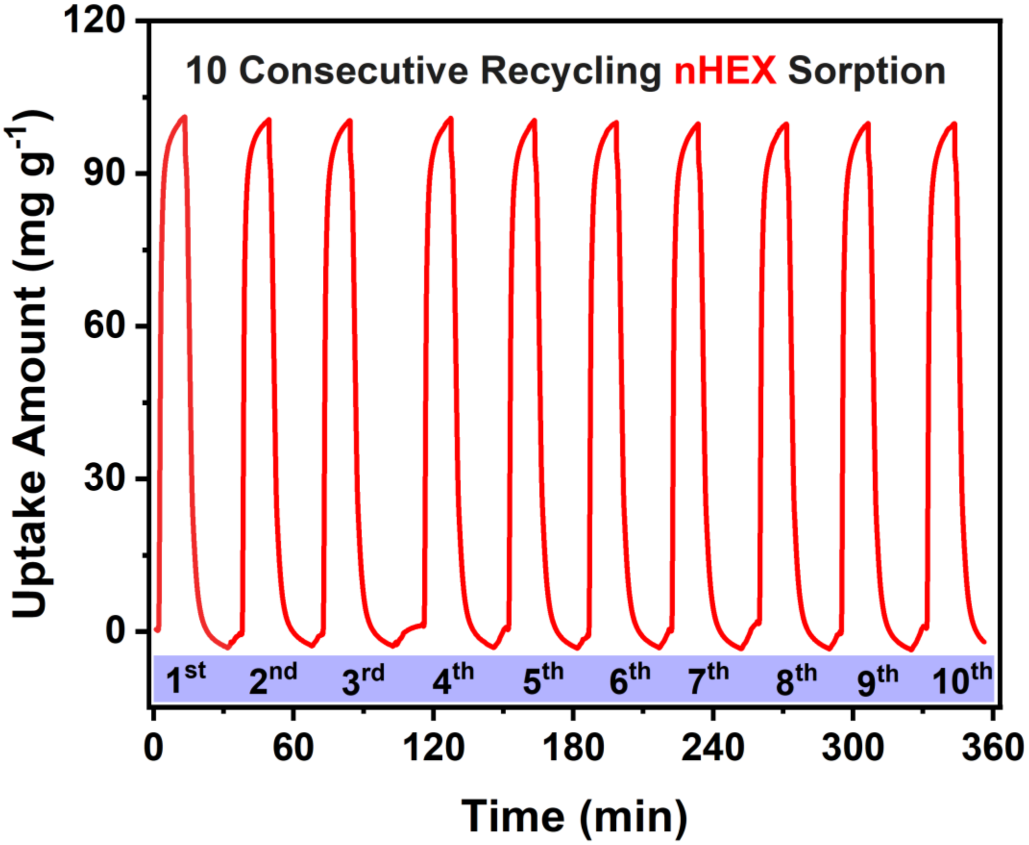

Figure S8. Adsorption-desorption recyclability tests of nHEX on MIL-120 for 10 consecutive sorption cycles at 120 °C.

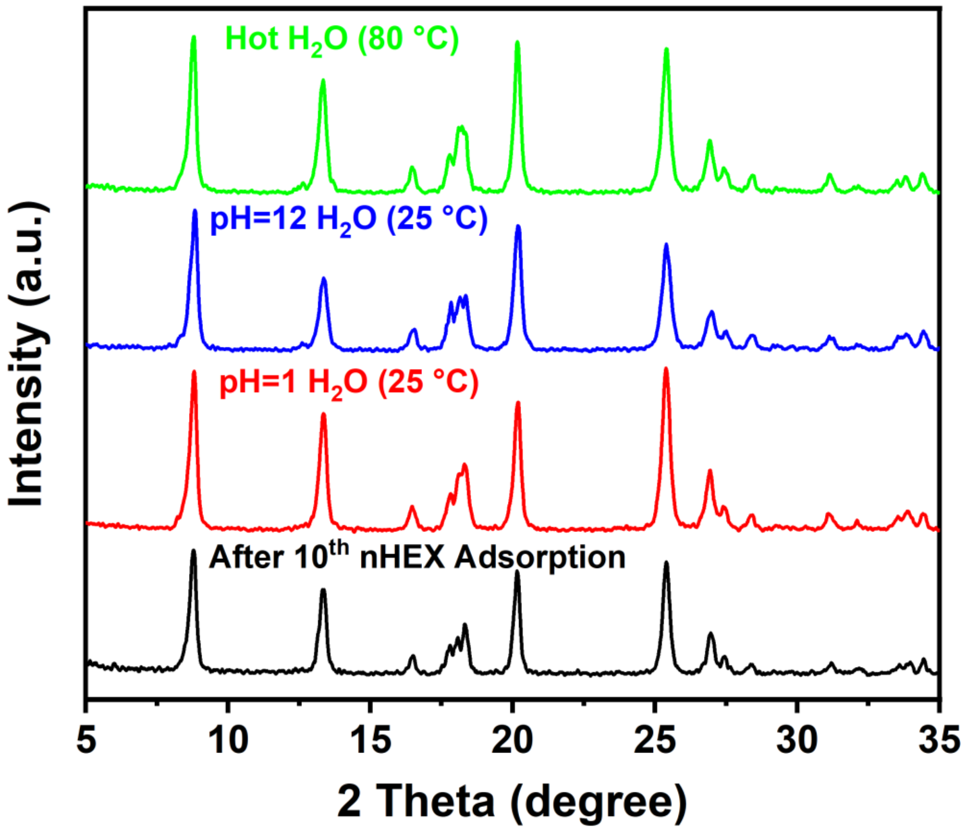

Figure S9. PXRD patterns of MIL-120 after exposing to multiple treatements and 10 consecutive sorption cycling.

Supporting Tables

Table S1. Comparison of physical properties of hexane isomers, including nHEX, 3MP, and 22DMB.[8]

Alkane Molecule	Boiling Point (K)	Kinetic Diameter (Å)	Research Octane Number
nHEX	341.6-342.2	4.5	24.8
3MP	336.0-336.8	5.0-5.5	74.5
22DMB	322.8-323.0	6.2	91.8

Table S2. Binding energies and diffusion barriers of hexane isomers in MIL-120.

Alkane Molecule	Binding Energy (eV)	Diffusion Barrier (eV)
nHEX	1.43	0.05
3MP	1.36	0.26
22DMB	0.97	0.77

Table S3. Summary of adsorption capacity of hexane isomers on various MOFs.

MOFs	nHEX Uptake (mg g^{-1})	3MP Uptake (mg g^{-1})	22DMB Uptake (mg g^{-1})	*T* (°C)	Ref.
MIL-120	135	87	17	30	This work
MIL-120	111	7.5	1.8	120	
$Ni(4\text{-}PyC)_2$	146	79	12	30	[9]
CAU-21-ODB	141	18	25	30	[10]
CAU-10-H	134	94	--	30	[11]
CAU-10-Br	47	11	4	30	[11]
Al-bttotb	150	94	10	30	[12]
Ca-tcpb	139	132	--	60	[13]
Ca-tcpb	98	11	--	120	[13]
Zn-adtb	120	20	7	30	[14]
Ni-Asp	128	18	14	30	[15]
Ni-Asp	70	12	10	150	[15]
Zr-bptc	130	25	8	150	[16]
Zr-abtc	110	87	50	150	[16]
PTA	176	9	--	50	[17]
$Fe_2(BDP)_3$	123	129	136	130	[18]
$Fe_2(BDP)_3$	112	108	116	160	[18]
Co-FA	128	136	24	30	[19]
UU-200	145	98	--	30	[20]
Mn-DHBQ	149	148	107	30	[21]
Mn-DHBQ	131	51	4	120	[21]
HIAM-203	142	123	--	30	[22]
HIAM-203	82	2	--	150	[22]
HIAM-318	126	115	19	30	[23]
HIAM-341	165	54	3	30	[24]
HIAM-341	113	50	0.5	100	[24]
HIAM-410	121	111	103	30	[25]
HIAM-601	110	9	2	30	[26]
HIAM-802	165	120	8	30	[26]

References

(1) Kresse, G.; Furthmüller, J. Efficient Iterative Schemes for ab initio Total-Energy Calculations Using A Plane-Wave Basis Set. *Phys. Rev. B* **1996**, *54*, 11169.
(2) Kresse, G.; Joubert, D. From Ultrasoft Pseudopotentials to The Projector Augmented-Wave Method. *Phys. Rev. B* **1999**, *59*, 1758.
(3) Thonhauser, T.; Cooper, V. R.; Li, S.; Puzder, A.; Hyldgaard, P.; Langreth, D. C. Van der Waals Density Functional: Self-Consistent Potential and The Nature of The van der Waals Bond. *Phys. Rev. B* **2007**, *76*, 125112.
(4) Berland, K.; Cooper, V. R.; Lee, K.; Schröder, E.; Thonhauser, T.; Hyldgaard, P.; Lundqvist, B. I. Van der Waals Forces in Density Functional Theory: A Review of The vdW-DF Method. *Rep. Prog. Phys.* **2015**, *78*, 066501.
(5) Thonhauser, T.; Zuluaga, S.; Arter, C.; Berland, K.; Schröder, E.; Hyldgaard, P. Spin Signature of Nonlocal Correlation Binding in Metal-Organic Frameworks. *Phys. Rev. Lett.* **2015**, *115*, 136402.
(6) Langreth, D.; Lundqvist, B. I.; Chakarova-Käck, S. D.; Cooper, V.; Dion, M.; Hyldgaard, P.; Kelkkanen, A.; Kleis, J.; Kong, L.; Li, S. A Density Functional for Sparse Matter. *J. Phys. Condens. Matter* **2009**, *21*, 084203.
(7) Henkelman, G.; Uberuaga, B. P.; Jónsson, H. A Climbing Image Nudged Elastic Band Method for Finding Saddle Points and Minimum Energy Paths. *J. Chem. Phys.* **2000**, *113*, 9901-9904.
(8) Xie, F.; Yu, L.; Wang, H.; Li, J. Metal-Organic Frameworks for C6 Alkane Separation. *Angew. Chem. Int. Ed.* **2023**, *62*, e202300722.
(9) Xie, F.; Yu, L.; Jenkins, T.; Shutak, J.; Tan, K.; Thonhauser, T.; Wang, H.; Li, J. Nickel Isonicotinate Framework with Optimal Pore Structure for Complete Discrimination of Hexane Isomers. *ACS Materials Lett.* **2023**, *6*, 43-48.
(10) Yu, L.; Ullah, S.; Yao, J.; Lin, D.; Huang, J.; Tu, S.; Luo, H.; Xia, Q.; Thonhauser, T.; Wang, H. Full Exclusion of Branched Hexanes from Their Linear Isomer by a Robust Aluminum Metal–Organic Framework with Tailored Pore Structure. *ACS Materials Lett.* **2023**, *5*, 1532-1536.
(11) Yu, Q.; Guo, L.; Lai, D.; Zhang, Z.; Yang, Q.; Yang, Y.; Ren, Q.; Bao, Z. A Pore-Engineered Metal-Organic Framework with Mixed Ligands Enabling Highly Efficient Separation of Hexane Isomers for Gasoline Upgrading. *Sep. Purif. Technol.* **2021**, *268*, 118646.
(12) Yu, L.; Dong, X.; Gong, Q.; Acharya, S. R.; Lin, Y.; Wang, H.; Han, Y.; Thonhauser, T.; Li, J. Splitting Mono-and Dibranched Alkane Isomers by a Robust Aluminum-Based Metal–Organic Framework Material with Optimal Pore Dimensions. *J. Am. Chem. Soc.* **2020**, *142*, 6925-6929.
(13) Wang, H.; Dong, X.; Velasco, E.; Olson, D. H.; Han, Y.; Li, J. One-of-a-kind: A Microporous Metal–Organic Framework Capable of Adsorptive Separation of Linear, Mono-and Di-branched Alkane Isomers via Temperature-and Adsorbate-Dependent Molecular Sieving. *Energy Environ. Sci.* **2018**, *11*, 1226-1231.
(14) Velasco, E.; Xian, S.; Wang, H.; Teat, S. J.; Olson, D. H.; Tan, K.; Ullah, S.; Osborn Popp, T. M.; Bernstein, A. D.; Oyekan, K. A.; et al. Flexible Zn-MOF with Rare Underlying scu Topology for Effective Separation of C6 Alkane Isomers. *ACS Appl. Mater. Interfaces* **2021**, *13*, 51997-52005.

(15) Chen, R.; Zhou, F.; Sheng, B.; Zhang, Z.; Yang, Q.; Yang, Y.; Ren, Q.; Bao, Z. Robust Nickel Aspartate Framework for Shape Recognition of Hexane Isomers. *ACS Sustain. Chem. Eng.* **2022**, *10*, 11330-11337.
(16) Wang, H.; Dong, X.; Lin, J.; Teat, S. J.; Jensen, S.; Cure, J.; Alexandrov, E. V.; Xia, Q.; Tan, K.; Wang, Q.; et al. Topologically Guided Tuning of Zr-MOF Pore Structures for Highly Selective Separation of C6 Alkane Isomers. *Nat. Commun.* **2018**, *9*, 1745.
(17) Chen, Q.; Xian, S.; Dong, X.; Liu, Y.; Wang, H.; Olson, D. H.; Williams, L. J.; Han, Y.; Bu, X. H.; Li, J. High-Efficiency Separation of n-Hexane by A Dynamic Metal-Organic Framework with Reduced Energy Consumption. *Angew. Chem. Int. Ed.* **2021**, *60*, 10593-10597.
(18) Herm, Z. R.; Wiers, B. M.; Mason, J. A.; van Baten, J. M.; Hudson, M. R.; Zajdel, P.; Brown, C. M.; Masciocchi, N.; Krishna, R.; Long, J. R. Separation of Hexane Isomers in A Metal-Organic Framework with Triangular Channels. *Science* **2013**, *340*, 960-964.
(19) Wang, H.; Dong, X.; Ding, J.; Wang, K.; Yu, L.; Zhang, S.; Han, Y.; Gong, Q.; Ma, A.; Li, J. Upgrading Octane Number of Naphtha by a Robust and Easily Attainable Metal-Organic Framework through Selective Molecular Sieving of Alkane Isomers. *Chem. Eur. J.* **2021**, *27*, 11795-11798.
(20) Zhang, Z.; Peh, S. B.; Kang, C.; Yu, K.; Zhao, D. Efficient Splitting of Alkane Isomers by a Bismuth-Based Metal-Organic Framework with Auxetic Reentrant Pore Structures. *Angew. Chem. Int. Ed.* **2022**, *61*, e202211808.
(21) Chen, R.; Li, L.; Olson, D. H.; Guo, L.; Chen, L.; Yang, Q.; Xu, Q.; Zhang, Z.; Ren, Q.; Li, J.; et al. Sequential Separation of Linear, Mono-, and Di-Branched Hexane Isomers on a Robust Coordination Polymer with Nonbonding Flexibility. *Small* **2023**, *19*, 2207367.
(22) Lin, Y.; Yu, L.; Ullah, S.; Li, X.; Wang, H.; Xia, Q.; Thonhauser, T.; Li, J. Temperature-Programmed Separation of Hexane Isomers by a Porous Calcium Chloranilate Metal-Organic Framework. *Angew. Chem. Int. Ed.* **2022**, *61*, e202214060.
(23) Zhou, X.; Ma, L. L.; Yu, L.; Zhou, K.; Xiong, K.; Gai, Y.; Li, J.; Wang, H. Size-Exclusion Separation of Hexane Isomers by a Y-MOF Built on {Y(COO)3}n Chains. *ACS Materials Lett.* **2024**, *6*, 928-932.
(24) Li, S.; Yu, L.; Zhou, X.; Zhou, K.; Yin, L.; Gao, M.; Wang, H. Molecular Sieving Separation of Hexane Isomers by an Aluminum-Based Metal-Organic Framework with nia Topology. *Chin. J. Chem.* **2025**, *43*, 2637-2641.
(25) Guo, F.-A.; Wang, J.; Chen, C.; Dong, X.; Li, X.; Wang, H.; Guo, P.; Han, Y.; Li, J. Linker Vacancy Engineering of a Robust ftw-type Zr-MOF for Hexane Isomers Separation. *Angew. Chem. Int. Ed.* **2023**, *62*, e202303527.
(26) Yu, L.; Li, S.; Zhou, X.; Zhang, B.; Zhou, K.; Xia, Q.; Wang, S.; Li, J.; Wang, H. Building Ultramicroporous Zirconium Metal–Organic Frameworks with Ligands of High Coordination Density through A Reticular Approach. *Nat. Chem.* **2025**, *17*, 1207-1215.
